# Ultrasound-guided obturator nerve block technique at the distal end of the obturator canal: case series and cadaver evaluation

**DOI:** 10.1007/s00540-024-03434-1

**Published:** 2024-12-02

**Authors:** Takayuki Yoshida, Yousuke Nakano, Masaaki Kitada, Tatsuo Nakamoto

**Affiliations:** 1 Department of Anesthesiology, Kansai Medical University Medical Center, 10-15 Fumizono-Cho, Moriguchi, Osaka 570-8507 Japan; 2 Department of Anatomy, Faculty of Medicine, Kansai Medical University, Hirakata, Osaka Japan; 3 Center for Clinical Anatomy, Kansai Medical University, Hirakata, Osaka Japan; 4 Department of Anesthesiology, Kansai Medical University Hospital, Hirakata, Osaka Japan

**Keywords:** Obturator nerve block, Transurethral resection of bladder tumor, Hip articular branch, Obturator canal, Ultrasound

## Abstract

The obturator nerve variably gives off the anterior, posterior, and hip articular branches along its course; however, all branches invariably pass through the obturator canal. Herein, we describe our obturator nerve block technique, which promises to deliver local anesthetic directly into the obturator canal. We performed the obturator nerve block in six patients undergoing transurethral resection of bladder tumor under spinal anesthesia. We also evaluated the spread of dye in a cadaver. With patient placed in the lithotomy position, a linear ultrasound transducer was placed on the anterior part of the genitofemoral sulcus, oriented cephalad, to observe the external orifice of the obturator canal, which is present immediately posterior to the posterior end of the inferior margin of the superior pubic ramus and just medial to the body of the ischium. The needle tip was guided to this location, and 10 ml of 1.5% lidocaine was injected into the obturator canal. The procedure was repeated using 10-ml dye in a cadaver. No obturator jerks were observed during the transurethral resection of bladder tumors in all six cases. Cadaver examination confirmed the dye stained the obturator nerve, with retrograde spread into the pelvic cavity via the obturator canal.

**Supplementary Information:**

The online version contains supplementary material available at 10.1007/s00540-024-03434-1.

## Introduction

The obturator nerve gives off the anterior, posterior, and hip articular branches variably in the pelvic cavity, obturator canal, or medial thigh [1]. Hence, spreading the local anesthetic solution into the obturator canal and further toward the pelvic cavity would be reasonable for blocking all nerve fibers derived from the obturator nerve. Several ultrasound-guided proximal approaches for obturator nerve block (ONB), with a single local anesthetic injection into the interfascial plane between the obturator externus and pectineus muscles, have been reported [2–6]. According to two cadaveric assessments [5, 7], dye administered into this interfascial plane shows a retrograde spread through the obturator canal toward the pelvic cavity. However, in a clinical study using contrast dye, obturator canal enhancement was observed in only 84% of participants, even when an ultrasound-guided proximal approach was used [8].

We previously reported an ultrasound-guided proximal ONB approach for patients in the lithotomy position [5]. The ultrasound transducer is placed on the genitofemoral sulcus, oriented cephalad, to observe the superior pubic ramus, obturator externus, and pectineus muscles. When performing this technique, we often observed on ultrasound images that the local anesthetic spread from the target interfascial plane through the area between the obturator externus muscle and superior pubic ramus toward the distal end of the obturator canal. Based on this finding, we developed an ultrasound-guided technique for ONB at the level of the obturator canal, with the assumption that this technique would be promising for delivering local anesthetics into the obturator canal. In this report, we describe our technique and its application in six patients undergoing transurethral resection of bladder tumor (TURBT). We also investigated the spread of the dye produced using our technique in a cadaver.

## Methods

This case series included six patients undergoing the transurethral resection of bladder tumors located on the lateral bladder wall at Kansai Medical University (KMU) Medical Center (Moriguchi, Japan) under spinal anesthesia and a unilateral ONB (Table 1). All patients provided written consent for the anesthesia method and surgery, after discussing the potential risks and benefits. The KMU Medical Center Research Ethics Board grants exemption of ethical approval for case series. Written informed consent was obtained from all six patients for the publication of this report.

**Table 1:** Patient characteristics

Cases	Sex	Age, years	Height, cm	Weight, kg	Block side	Operative time, min	Bupivacaine dose, ml	Procedure time, min
1	M	79	171	69	R	18	3.0	14
2	M	82	162	72	R	98	3.0	9
3	M	76	179	55	R	33	3.5	5
4	M	82	157	63	R	69	3.2	5
5	M	80	167	62	L	41	3.0	15
6	F	77	157	52	L	30	3.0	9

Block side indicates the side receiving the obturator nerve block. Bupivacaine (isobaric, 0.5%) was used for spinal anesthesia. Procedure time was derived as the time between the completion of spinal anesthesia and the completion of obturator nerve block, including the patient’s positional change

*M* male, *F* female

**Fig. 1:**
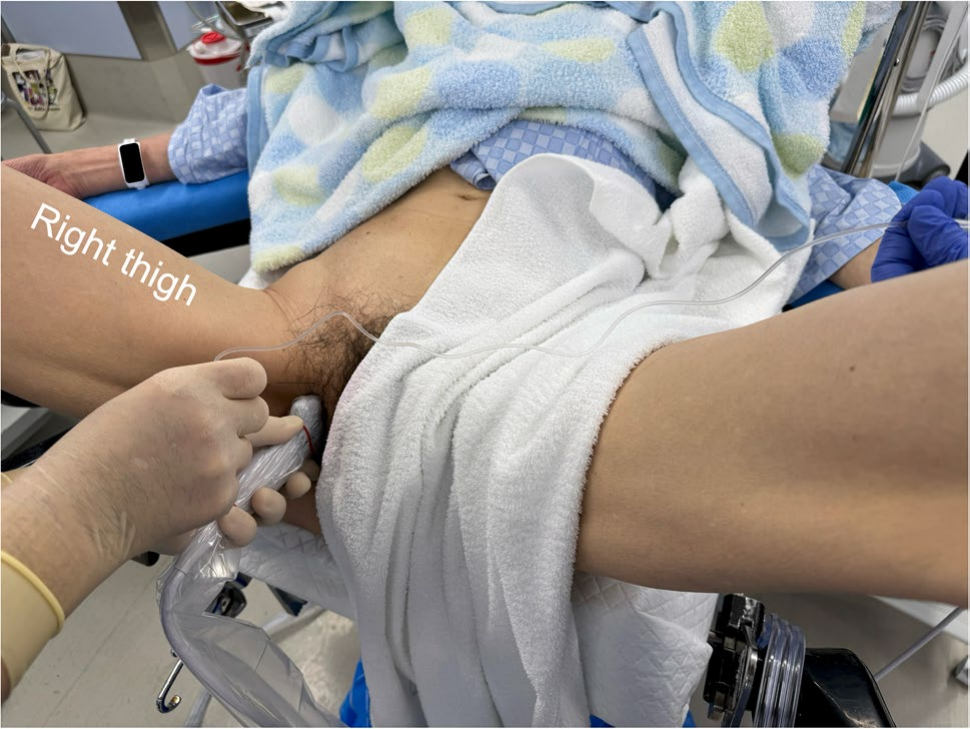
Performance of a right-side obturator nerve block, with the patient in the lithotomy position

**Fig. 2:**
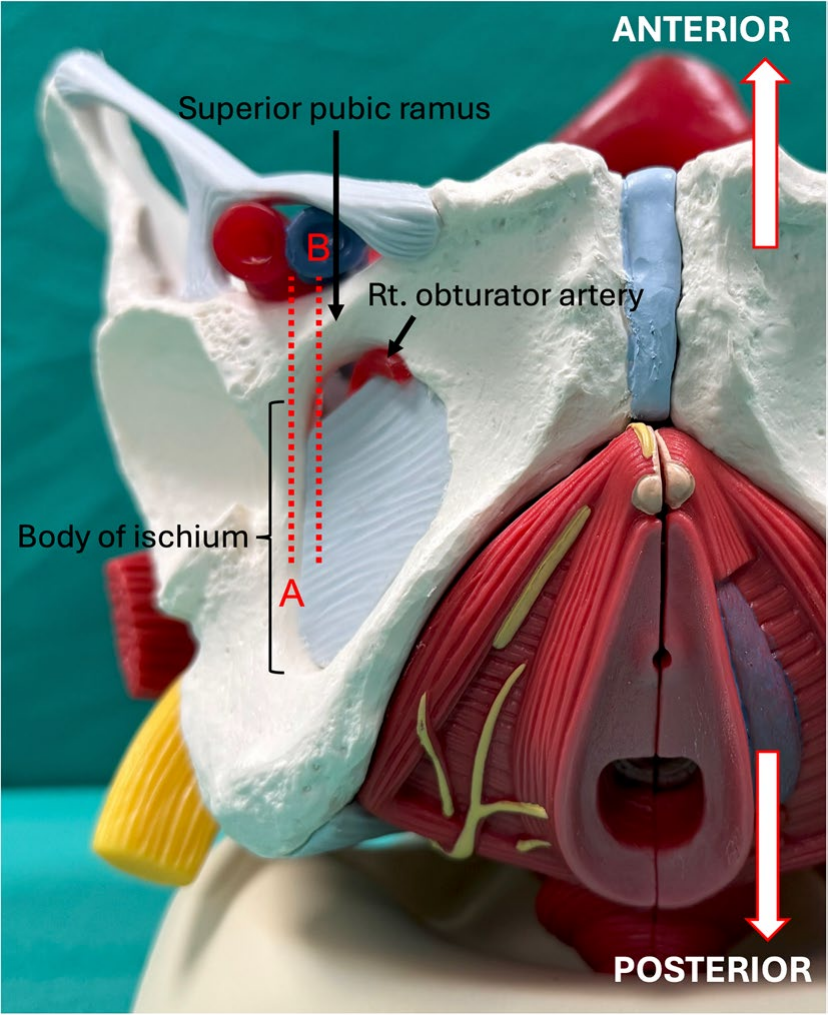
Scanning planes are shown, with reference lines, as follows: dotted line A, the level to see the body of ischium; and dotted line B, the level to see the external orifice of the obturator canal

**Fig. 3:**
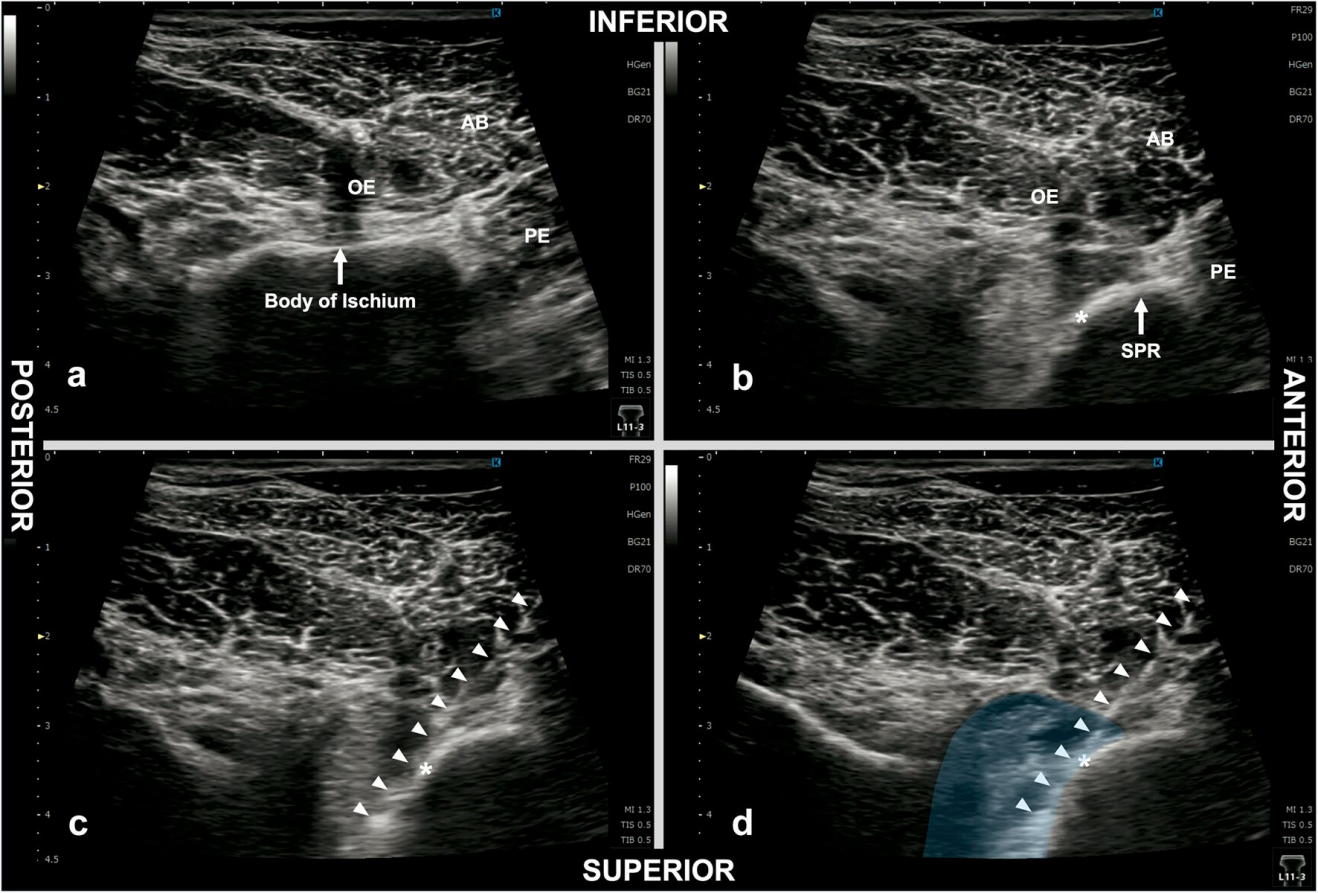
Ultrasound images obtained during the obturator nerve block procedure. **a** The pre-procedure view obtained at the level indicated by line A, as shown in Fig. 2. The hyperechoic line with an acoustic shadow, seen deep (superior) to the obturator externus muscle, indicates the body of ischium. **b** The pre-procedure view obtained at the level indicated by line B, as shown in Fig. 2. The external orifice of the obturator canal is located immediately posterior to the posterior end point (asterisk) of the inferior margin of the superior pubic ramus. **c** The needle trajectory to the obturator canal (arrow heads). The needle tip is positioned deep (superior) to the level of the inferior margin of the superior pubic ramus and posterior to the superior pubic ramus. **d** The image taken after injection of 10-ml local anesthetic. Local anesthetic (blue-shaded area) spreads toward the obturator canal. *AB* Adductor brevis muscle, *OE* Obturator externus muscle, *PE* Pectineus muscle, *SPR* Superior pubic ramus

After establishing standard monitoring, patients were placed in the left lateral decubitus position. Then, an anesthesiologist performed spinal anesthesia at L2/3 or L3/4 intervertebral space using a 25-G Quincke spinal needle and 3.0–3.5 ml of isobaric 0.5% bupivacaine. After confirming sufficient distribution of the spinal anesthesia in the supine position, the patient was placed in the lithotomy position. A high-frequency linear ultrasound transducer (L11-3, KONICA MINOLTA, Tokyo, Japan), connected to an ultrasound apparatus (SONIMAGE MX1, KONICA MINOLTA), was placed over the anterior part of the genitofemoral sulcus, defined as the boundary between the perineum and medial thigh, on the side the bladder tumor was present (Fig. 1). The transducer was then tilted slightly laterally to observe the body of the ischium (scanning plane indicated by Line A in Fig. 2), which was seen as a flat hyperechoic line with an acoustic shadow (Fig. 3a). Subsequently, the transducer was tilted back in the medial direction until tissue with no acoustic shadow appeared posterior to the superior pubic ramus (scanning plane indicated by Line B in Fig. 2). The distal end of the obturator canal is located in this area, posterior to the superior pubic ramus, deep to the obturator externus muscle (Fig. 3b). We confirmed that the obturator artery was not observed on the predicted needle trajectory using the color Doppler function before commencing needle insertion. After disinfection around the expected puncture site, an 80 mm, 22 G block needle (Stimuplex Ultra 360; B. Braun, Melsungen, Germany) was inserted, approximately 1.0 cm anterior to the anterior end of the transducer (Fig. 1). The needle was advanced in plane with the transducer under ultrasound guidance; the needle tip was placed at the distal end of the obturator canal, which, as per our observations, is present immediately posterior to the posterior end point (indicated by an asterisk in Fig. 3) of the inferior margin of the superior pubic ramus on ultrasound (Fig. 3c). After negative aspiration for blood, 10 ml of 1.5% lidocaine was administered (Fig. 3d, Supplementary Video 1). Subsequently, urologists commenced TURBT using bipolar diathermy.

The cadaveric assessment was implemented as part of the KMU Cadaver Surgical Training program, held from February 11 to 12, 2024 (KMU Research Ethics Committee approval number: 2023261). A female adult cadaver embalmed using Thiel’s method was used for the assessment [9, 10]. Using the approach described above, one of the authors (TY) placed an 18-G Tuohy needle tip at the distal end of the obturator canal on the left side of the cadaver with its left hip flexed and externally rotated. Subsequently, 10 ml of blue, water-soluble dye was injected. Approximately, 1 h after the dye injection, the left thigh and pelvis were dissected to evaluate the dye distribution.

## Results

**Fig. 4:**
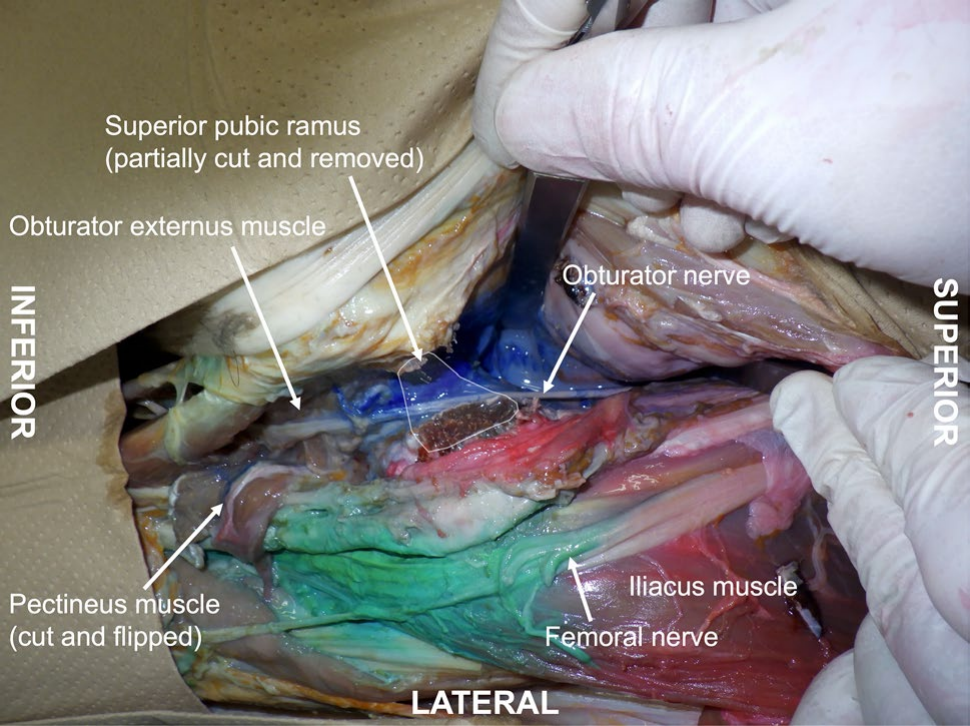
Spread of blue dye (10 ml) in a cadaveric specimen after left-sided ultrasound-guided obturator nerve block. A retrograde spread of the blue dye is observed, from the intermuscular space between the obturator externus and pectineus muscles to the pelvic cavity through the obturator canal, staining the obturator nerve. Note that the superior pubic ramus has been partially removed (space enclosed by the white line) to view the obturator canal. The figure is reprinted with permission from the Kansai Medical University Cadaver Surgical Training program (Department of Anatomy, Faculty of Medicine, Kansai Medical University, Hirakata, Japan). Due to the nature of this training program, femoral nerve block and pericapsular nerve group block procedures using 10 ml of green and red dyes, respectively, have also been performed

The TURBT was successfully completed in all cases. There was no occurrence of obturator jerk during surgery and no complications, such as vessel puncture, hematoma, or nerve injury, resulting from the ONB. Sensory and motor blocks produced by the ONB were not assessed in all patients as spinal anesthesia was expected to be in effect for longer than ONB. In the cadaveric evaluation, the blue dye stained the obturator nerve, with retrograde spread from the distal end of the obturator canal into the pelvic cavity (Fig. 4).

## Discussion

Herein, we describe a new ultrasound-guided ONB approach performed in a case series of six patients undergoing TURBT, with a cadaveric investigation. We confirmed that the needle tip can be placed in the distal end of the obturator canal under ultrasound guidance with patients in the lithotomy position.

Anagnostopoulou et al. [1] reported that bifurcation of the obturator nerve into the anterior and posterior branches variably occurs within the pelvic cavity, obturator canal, and medial thigh in 23.22%, 51.78%, and 25% of the specimens, respectively. In some specimens, the posterior branch passes through the obturator externus muscle immediately after emerging from the obturator canal, which means that it does not exist between the obturator externus and pectineus muscles [1, 8]. Moreover, the articular branches to the hip joint arise from the common obturator nerve or its divisions at various levels [1]. Therefore, the articular branches do not always lie between the obturator externus and pectineus muscles. Consequently, constraining local anesthetic infiltration only to the intermuscular space, the target of previously reported ultrasound-guided proximal ONB approaches, does not guarantee blockade of the posterior and hip articular branches of the obturator nerve. When the local anesthetic fills the obturator canal, the obturator nerve, including its anterior and posterior divisions and the hip articular branches, are blocked given that all these nerve fibers unexceptionally run through the obturator canal [1, 11]. When 10 ml of the solution was injected at the distal end of the obturator canal using our approach, we noted that only a small amount of the local anesthetic solution overflowed from the obturator canal, as seen in Supplementary Video 1. This implied that most of the injected local anesthetic entered the obturator canal, as predicted and confirmed in our cadaveric examination.

A potential advantage of our current approach over our previous proximal ONB technique [5] would be the feasibility of directly delivering local anesthetic into the obturator canal.

Therefore, our current approach to filling the obturator canal with local anesthetic may reduce the required local anesthetic volume compared to previously reported proximal approaches. Nevertheless, a dose-finding study is needed to confirm this postulation. To ensure a wide safety margin, we determined the use of 10 ml of local anesthetic, as used in previous research regarding the conventional proximal ONB approach [8]. Concerning possible disadvantages, the angle between the needle trajectory and ultrasound beams is smaller with our current approach than with our previous approach, leading to less needle visibility under ultrasound guidance.

Our study has some limitations. Our findings are based on only six cases and one cadaveric examination. Moreover, we did not specifically assess the effects of the ONB, although this study aimed to demonstrate the feasibility of seeing the obturator canal, placing the needle tip at the external orifice of the canal under ultrasound guidance, and performing an ONB. Furthermore, we did not employ nerve stimulation to locate the obturator nerve. Since all nerve fibers arising from the obturator nerve run through the obturator canal [1, 11], injecting a local anesthetic into the obturator canal should produce the ONB without the need for locating the nerve. However, for safety reasons, we should have combined nerve stimulation with our new ultrasound-guided ONB technique, because the movement of the adductor muscles must be controlled during TURBT. Nerve stimulation can also be helpful to avoid direct obturator nerve injury during needle advancement.

In summary, this case series, combined with a cadaver examination, provides a good foundation for future studies to confirm the usefulness of our ultrasound-guided obturator canal approach for ONB.

## Supplementary Information

Below is the link to the electronic supplementary material.

Supplementary file1 (MP4 49575 KB)

## Data Availability

Data are available upon reasonable request.
